# Carbonagogue Medication

**Published:** 1851-06

**Authors:** 


### CARBONAGOGUE MEDICATION.

The monograph on this subject in the present number of this *Journal*, from the pen of Professor L. Rogers, will arrest the attention of readers, and will richly repay perusal and study. The various questions belonging to the theme–carbonagogue medication are handled by Dr. Rogers with a great deal of skillful power, and few can rise from the study of the paper, without a feeling of substantial benefit. The path of investigation, on which Dr. Rogers started, could not fail, under his method of travelling, to lead to results eminently practical and useful, and the practitioner who may apply the principles set forth by Dr. Rogers will find a gratifying success

We have rarely read anything in medical literature, of a practical character, that gave us the same degree of satisfaction, that we have derived from the perusal of this paper on "carbonagogue medication." As a contribution to therapeutical science, we know nothing superior to it. We have often verified in practice the truths set forth by Dr. Rogers, and the investigations, and subjects for observation indicated by him, open a wide range, in which a properly constituted mind may reap a rich harvest of reputation. B.

